# Novelty and Dopaminergic Modulation of Memory Persistence: A Tale of Two Systems

**DOI:** 10.1016/j.tins.2018.10.002

**Published:** 2019-02

**Authors:** Adrian J. Duszkiewicz, Colin G. McNamara, Tomonori Takeuchi, Lisa Genzel

**Affiliations:** 1 Montreal Neurological Institute and Hospital, McGill University, Montreal, Canada; 2 Centre for Discovery Brain Sciences, University of Edinburgh, Edinburgh, UK; 3 MRC Brain Network Dynamics Unit, Department of Pharmacology, University of Oxford, Oxford, UK; 4 Danish Research Institute of Translational Neuroscience (DANDRITE), Nordic-EMBL Partnership for Molecular Medicine, Aarhus University, Aarhus, Denmark; 5 Department of Biomedicine, Aarhus University, Aarhus, Denmark; 6 Aarhus Institute of Advanced Studies (AIAS), Aarhus University, Aarhus, Denmark; 7 Donders Institute for Brain, Cognition, and Behaviour, Radboud University and Radboudumc, Nijmegen, The Netherlands

**Keywords:** dopamine, novelty, memory consolidation, hippocampus, episodic memory, semantic memory

## Abstract

Adaptation to the ever-changing world is critical for survival, and our brains are particularly tuned to remember events that differ from previous experiences. Novel experiences induce dopamine release in the hippocampus, a process which promotes memory persistence. While axons from the ventral tegmental area (VTA) were generally thought to be the exclusive source of hippocampal dopamine, recent studies have demonstrated that noradrenergic neurons in the locus coeruleus (LC) corelease noradrenaline and dopamine in the hippocampus and that their dopamine release boosts memory retention as well. In this opinion article, we propose that the projections originating from the VTA and the LC belong to two distinct systems that enhance memory of novel events. Novel experiences that share some commonality with past ones (‘common novelty’) activate the VTA and promote semantic memory formation via systems memory consolidation. By contrast, experiences that bear only a minimal relationship to past experiences (‘distinct novelty’) activate the LC to trigger strong initial memory consolidation in the hippocampus, resulting in vivid and long-lasting episodic memories.

## Highlights

Novelty induces dopamine release in the hippocampus, triggering memory consolidation to boost memory persistence.

Two dopaminergic systems (the ventral tegmental area- and locus coeruleus-hippocampus systems) can stabilise memory through novelty-induced dopamine release in the hippocampus.

Novel experiences can be viewed as a spectrum, from experiences that, while clearly novel, share some commonality with past experiences (‘common novelty’), to more fundamentally distinct experiences that bear minimal relationships to past experiences (‘distinct novelty’).

We propose that events characterised by ‘common novelty’ boost memory retention via activation of the ventral tegmental area-hippocampus system, resulting in initial consolidation followed by systems consolidation to create neocortical, semantic, long-term memories.

We further propose that events characterised by ‘distinct novelty’ lead to the boost of detailed hippocampal, episodic, long-term memory via activation of the locus coeruleus-hippocampus system through strong upregulation of the synaptic tagging and capture mechanism.

## Two Origins of Hippocampal Dopamine

Dopaminergic neuromodulation plays diverse roles in the central nervous system, depending largely on its source and the target brain areas. In addition to its well-known role in influencing ongoing and future behaviour through the control of movement 1, 2 and reward signalling 3, 4, 5, it has also been suggested as a critical modulator of hippocampal-dependent mnemonic processes, acting to selectively enhance retention at different stages of memory formation 6, 7. Behavioural studies have confirmed that activation of **dopamine D_1_/D_5_ receptors** (see Glossary) not only contributes to memory encoding [8] but is also necessary to convert short-term memory to protein synthesis-dependent long-term memory 9, 10, 11, 12, 13. Although dopamine D_2_-like receptors (D_2_, D_3_, and D_4_) are also expressed in the hippocampus and their activation has been shown to affect hippocampal plasticity and excitability 14, 15, 16, 17, 18, 19, their role in memory consolidation processes has not yet been thoroughly characterized.

**Figure 1:**
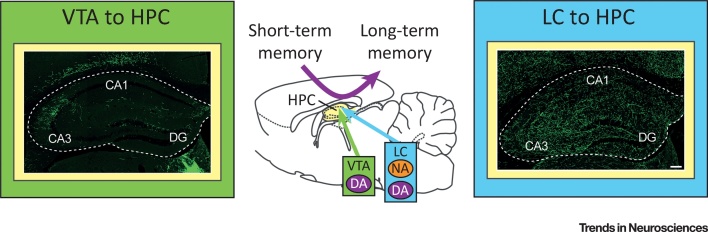
Two Dopaminergic Systems for Memory Consolidation. Both the ventral tegmental area (VTA; labelled green) and locus coeruleus (LC; cyan) project to the dorsal hippocampus in mice, but projections from LC (right panel) are denser than those from VTA (left). Both VTA and LC neurons can promote memory persistence via dopamine (DA) D_1_/D_5_ receptor-dependent mechanisms in the hippocampus and thus presumably via direct release of dopamine from their axons. Reproduced from [28].

This enhancement of memory retention is achieved by prolonging the stability of changes in synaptic efficacy 12, 20, most notably long-term potentiation (LTP), a neural substrate for memory storage (for review see 21, 22; see also Box 1). Novel experiences have been shown to induce dopamine release in the hippocampus, promoting encoding and persistence of transient memory traces on the physiological as well as behavioural level 10, 12, 23, 24. The source of hippocampal dopamine was initially assumed to be the hippocampal terminals originating from **tyrosine-hydroxylase expressing (TH^+^)** neurons in the **ventral tegmental area (VTA)** 6, 25, 26. Indeed, optogenetic activation of this projection was shown to modulate hippocampal synaptic responses [19] and to enhance memory retention [27]. However, recent studies utilising *ex vivo* and *in vivo* approaches have collectively demonstrated that TH^+^ neurons in the **locus coeruleus (LC)** are also capable of enhancing memory retention through corelease of dopamine, in addition to noradrenaline, in the hippocampus 28, 29, 30. Although both dopaminergic projections in the dorsal hippocampus (Figure 1) promote **memory consolidation**, the reason for the existence of two separate dopaminergic inputs to the hippocampus is currently unknown. In this opinion article, we characterise the recent advances in our understanding of these two parallel dopaminergic systems and propose a framework for their predicted roles in different memory consolidation processes.

Box 1 **Initial Memory Consolidation within the Hippocampus**

In the first few hours after memory encoding, initial (or cellular) memory consolidation processes are required for memories to last 21, 87, 88. In absence of a neuromodulatory signal, hippocampal synapses modified during encoding via canonical NMDA (*N*-methyl-D-aspartate)-type glutamate receptor-mediated plasticity mechanisms return to their baseline state (Figure IA). However, heterosynaptic activation of metabotropic neuromodulatory receptors (most notably dopamine D_1_/D_5_ receptors) followed by *de novo* protein synthesis in the same neuronal population promotes persistence of these normally transient synaptic modifications, preventing the associated memory traces from being wiped out (Figure IB) (see [7] for review).

Importantly, dopamine D_1_/D_5_ receptor activation does not need to happen at the time of memory encoding. The synaptic tagging and capture mechanism 51, 52 enables hippocampal neurons to preserve synaptic modifications that happened within a few hours’ time window (or a ‘grace period’) around the time of dopaminergic activation. Neurons keep track of recently potentiated synapses with ‘synaptic tags’ induced by post-translational mechanisms (e.g., phosphorylation and actin dynamics, etc.) [89]. These tags promote the capture of ‘plasticity-related proteins (PRPs)’ that are synthesised *de novo* in response to activation of dopamine D_1_/D_5_ receptors. Once the potentiated and tagged synapse captures the PRPs, the LTP that would normally decay to baseline after several hours is instead transformed into a long-lasting, stable form. As both synaptic tags and PRPs have a life-span in the order of hours, the time-window of the availability of these two factors defines the ‘grace period’ for late-associativity. In other words, the tagged synapses that are potentiated within a few hours’ window before or after the dopamine D_1_/D_5_ receptor activation are consolidated. What follows is that the synaptic changes, even those of a normally transient nature, are preserved within the brain thanks to long-lasting plasticity within the associative hippocampal network.

This physiological phenomenon can be demonstrated on a behavioural level through an analogous phenomenon of ‘behavioural tagging’ 10, 12, 28, 66. In such experimental protocols, memory tasks inducing weak memory (that does not normally undergo initial memory consolidation) are coupled with unrelated novel events experienced close in time. If the two events occur within the ‘grace period’ postulated by the synaptic tagging and capture mechanism, the novel event boosts persistence of the weak memory via LC-mediated dopamine release and subsequent activation of hippocampal dopamine D_1_/D_5_ receptors [28]. Importantly, both synaptic tags induced by weak memory encoding and PRP production triggered by unrelated novel events have to happen in the same neuronal population, and sharing hippocampal neuronal ensembles between transient and novel/unexpected memories is a postulated network mechanism of the synaptic tagging and capture theory [68]. In the hippocampus, such overlap in neuronal ensembles representing events encoded close in time is achieved by increased neuronal excitability in a CREB (cAMP-responsive element-binding protein)-dependent manner 22, 90.

Alt-text: Box 1

**Figure I:**
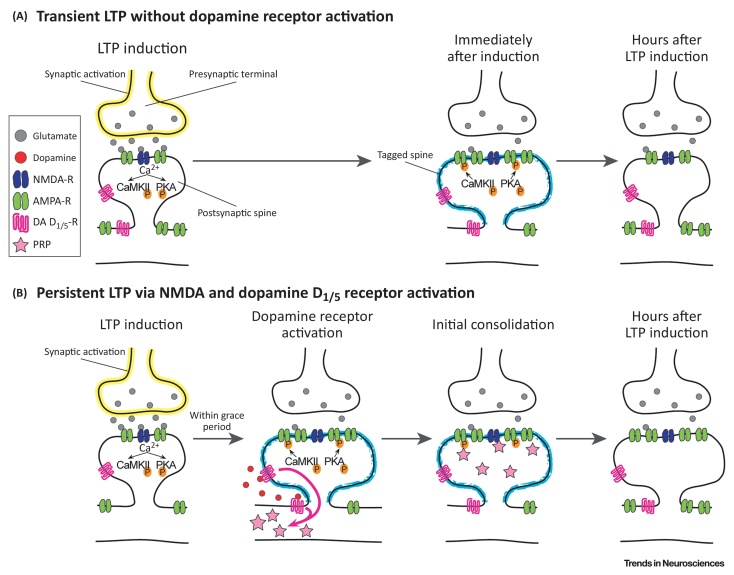
Dopamine D_1_/D_5_ Receptor Activation Promotes Initial Memory Consolidation within the Hippocampus. (A) *N*-methyl-d-aspartate (NMDA) receptor (NMDA-R) activation by presynaptic glutamate release coupled with postsynaptic depolarisation leads to transient long-term potentiation (LTP) of the synapse via Ca^2+^/calmodulin-dependent protein kinase II (CaMKII) and protein kinase A (PKA)–mediated α-amino-3-hydroxy-5-methyl-4-isoxazolepropionic acid (AMPA) receptor (AMPA-R) phosphorylation and their postsynaptic recruitment. The postsynaptic spine is also tagged for consolidation but in absence of plasticity-related proteins (PRPs) synaptic strength decays to baseline after several hours. (B) Activation of dopamine (DA) D_1_/D_5_ receptors (DA D_1/5_-R) leads to *de novo* PRP synthesis. If dopamine D_1_/D_5_ receptors are activated in the same neuron around the time of LTP induction, PRPs will be captured by the tagged synapses, leading to persistent LTP through initial consolidation.

## An Unexpected New Source of Dopamine

TH^+^ neurons in the midbrain, most notably in VTA, project sparsely to the hippocampus in rodents 25, 26, 27, 31 and potentially somewhat more densely in primates 32, 33. Nonetheless, the mismatch between the low density of VTA-TH^+^ axons (Figure 1) and high density of dopamine D_1_/D_5_ receptors in the rodent hippocampus (for review see [34]) has raised questions about the possibility of other sources of dopamine to the hippocampus [35]. Similarly, dopamine concentration reported in many neocortical areas does not seem to match the local density of axons from VTA-TH^+^ neurons [35]. Surprisingly, neocortical dopamine levels are strongly attenuated by activation of α_2_-adrenoceptors, a pharmacological manipulation that suppresses the noradrenergic system 35, 36. This led Paola Devoto and colleagues 35, 36 to suggest that LC-TH^+^ neurons, which project to many diverse brain areas and synthesise dopamine as a precursor for noradrenaline, may in fact be the dominant source of dopamine in some cortical regions. Like dopamine, noradrenaline serves an important role in synaptic plasticity [37] as well as mnemonic processes in the hippocampus 38, 39, 40 (Box 2).

Box 2 **Noradrenaline and Hippocampal Plasticity and Memory**

While recent behavioural studies highlighted the mnemonic role of dopamine released from LC-TH^+^ axons in the hippocampus 28, 29, 30, 41, it is important to emphasise that LC fibres are primarily known as the main source of noradrenaline in the central nervous system. Adrenoceptors are expressed ubiquitously in the hippocampus [91] and a large body of work focused on characterising their importance in hippocampal plasticity and memory 92, 93, 94, 95, 96, 97. Pharmacological blockade of β-adrenoceptors has been shown to impair long-term plasticity in the dentate gyrus in a manner similar to the role of dopamine D_1_/D_5_ receptors in CA1 98, 99. Moreover, activation of LC enhances synaptic plasticity in the dentate gyrus in β-adrenoceptor-dependent manner 100, 101, indicating that noradrenaline and dopamine may act in concert to strengthen hippocampal memory traces.

Behavioural studies utilising pharmacological blockade of hippocampal β-adrenoceptors in rats observed robust impairment in memory consolidation 38, 40, 102, 103 and in LC-mediated enhancement of memory encoding [101], establishing a link between noradrenergic modulation of hippocampal plasticity and memory. However, recent studies in mice did not detect interference of contextual memory encoding [30] or attenuation of LC-TH^+^ neuron-mediated boost in memory encoding [29] and consolidation [28] in response to similar pharmacological interventions. This discrepancy warrants further investigation and may be due to different behavioural tasks, differences between animal species, or the timing of β-adrenoceptor blockade.

Alt-text: Box 2

Evidence of dopamine corelease from the axons of LC-TH^+^ neurons projecting to the hippocampus first came from indirect inference in both *ex vivo* and *in vivo* experiments 41, 42. More specific evidence was subsequently provided by the demonstration that the memory consolidation-boosting effect created by optogenetic activation of LC-TH^+^ neurons was prevented through a pharmacological blockade of hippocampal dopamine D_1_/D_5_ receptors 28, 29, and, finally, by the direct detection of dopamine release after prolonged optogenetic stimulation of hippocampal LC-TH^+^ axons *ex vivo* [29].

In comparison with the projection by VTA-TH^+^ neurons, the LC-TH^+^ innervation of the hippocampus is more dense 28, 30. This suggests that hippocampal LC-TH^+^ terminals may mediate more global dopamine release while dopamine released from VTA-TH^+^ terminals could have a more specific effect, perhaps by selectively targeting particular interneuron types 19, 43. Still, direct quantitative comparisons of hippocampal dopamine release from LC-TH^+^ and VTA-TH^+^ axons are yet to be reported. Given that VTA-TH^+^ terminals are specialised for dopamine release whereas LC-TH^+^ terminals corelease dopamine and noradrenaline, it is conceivable that despite the differences in projection density, both projections are equally potent sources of hippocampal dopamine [44]. Furthermore, increased dopamine concentration was not detected *in vivo* after short phasic LC stimulation [39] which indicates that in contrast to intermittent, phasic LC activity reported in response to discrete stimuli 45, 46, 47 only a sustained increase in LC-TH^+^ neuron activity on the scale of minutes may induce dopamine corelease 28, 29. While further investigation is required to determine the exact dopamine release dynamics of each projection, it is possible that LC-TH^+^ terminals release minimal dopamine in response to moderate, transient LC activation but are then suited to releasing large amounts of dopamine in response to infrequently occurring sustained increases in LC activity. By contrast, dopamine release dynamics of VTA-TH^+^ terminals may be closer to linear, allowing downstream responses to subtler changes in VTA-TH^+^ neuron firing.

## Possible Roles for the Distinct Dopamine Systems in Memory Consolidation

Despite its modest innervation density, the VTA-hippocampus system is clearly functional both *ex vivo* and *in vivo* 19, 27, 48. Optogenetic activation of hippocampal VTA-TH^+^ axons can bidirectionally modulate CA3–CA1 synaptic responses *ex vivo* [19]. Photo-activation of hippocampal VTA-TH^+^ axons during spatial learning over many trials increases memory strength and promotes stability of the hippocampal spatial map as tested 1 h after memory encoding [27]. Similarly, optogenetic activation of hippocampal LC-TH^+^ axons during memory encoding promotes spatial memory retention [29] whereas inhibition of hippocampal LC-TH^+^ axons projecting specifically to the CA3 subregion during memory encoding blocks formation of new contextual memories and disrupts the stability of spatial representations in CA3 [30].

Notably, dopamine released by hippocampal LC-TH^+^ terminals creates a ‘grace period’ of enhanced memory persistence that can strengthen seemingly unrelated memory traces in a manner reminiscent of vivid ‘**flashbulb memories**’ reported in novel and surprising situations 49, 50. Evidence for this comes from selective manipulation of the initial (or cellular) memory consolidation phase (30 min after memory encoding). In this experiment, optogenetic activation of LC-TH^+^ but not VTA-TH^+^ neurons enhanced the persistence of seemingly unrelated spatial memories encoded close in time in the same way as a novel experience [28]. This interesting consequence of the activation of LC-hippocampal system is in line with the synaptic tagging and capture theory of initial memory consolidation, which explains such late-associativity of hippocampal memory traces by virtue of short-lived ‘synaptic tags’ present in recently potentiated synapses (Box 1) 51, 52. Moreover, because of a 30-min delay between memory encoding and optogenetic LC-TH^+^ neuron activation, this beneficial effect on memory persistence is unlikely due to noradrenaline-mediated changes in attention or arousal.

Activation of the VTA-hippocampal system, on the other hand, has been shown to promote the network level process known as **hippocampal reactivation** [27]. During sleep, neural patterns present at the time of preceding awake experiences are reactivated in a time-compressed manner via fast hippocampal oscillatory events called **sharp wave-ripples (SWRs).** This is important for the stabilisation of previously encoded memories and hippocampal representations 53, 54, 55. Hippocampal reactivation is associated with ‘systems memory consolidation’ that transforms new memories off-line during sleep, extracting overlapping content across multiple events (Box 3) 56, 57, and that involves coordinated activity between the hippocampus and the prefrontal cortex, mediated by SWRs 53, 54, 58, 59. SWRs promote the reinstatement of hippocampal representations for novel, but not familiar, places [55], indicating the importance of novelty in this consolidation process. Interestingly, dopamine released by hippocampal VTA-TH^+^ terminals at the time of the experienced event is thought to enhance systems memory consolidation. In support of this, optogenetic stimulation of hippocampal VTA-TH^+^ axons as the animal explores an environment with novel geometry enhances subsequent SWR-associated hippocampal reactivation [27]. Moreover, activation of this projection during encoding of a spatial route-learning task called the ‘crossword maze’ promotes memory persistence as measured through hippocampal place cell map stability as well as behavioural performance [27]. Interestingly, this boost in reactivation fidelity is limited to the activity patterns observed during the period of stimulation of hippocampal VTA-TH^+^ axons and does not extend to the patterns representing immediately preceding experiences [27], suggesting, at the physiological level, the VTA-hippocampal system is less suited to extending the ‘grace period’ of enhanced memory persistence.

Box 3 **Brain-Wide Network Reorganisation during Systems Memory Consolidation**

Due to the limited capacity of our memory systems, our brains need to decide which experiences to integrate into our long-term memory. Brain-wide reactivation of recent experience allows for the comparison of new memories with the experiences and knowledge already stored in the memory systems, thus perhaps leading to the integration of similar memories into pre-existing memory networks. The process whereby initially hippocampal-dependent memories become less dependent on the hippocampus and more dependent on the neocortex over time is referred to as ‘systems memory consolidation’ [60].

Events that can be incorporated into pre-existing networks are reactivated during subsequent sleep/rest [104] under neocortical guidance 105, 106 and are thus gradually integrated into neocortical networks [107]. In principle, this could be achieved by the mechanisms postulated early on by Marr [69] and modelled by McClelland and colleagues 70, 107. Memories are thought to be consolidated from the short-term, temporally organized storage of the hippocampus to a long-term neocortical, semantic memory network, becoming more categorised by content instead of time 60, 88, 108.

On a physiological level, systems memory consolidation is thought to integrate new memories into pre-existing neocortical networks during sleep/rest through coordinated reactivation of cell assemblies [57]. This bidirectional communication between the hippocampus and prefrontal cortex might be orchestrated through slow oscillations and hippocampal SWRs; slow oscillations travel from the neocortex to the hippocampus to first induce SWR-associated hippocampal reactivation, and subsequently reactivation in the neocortex time-locked to SWRs 58, 109, 110, 111. This sequential reactivation is thought to lead to the strengthening of neocortical connections and integration of new information into pre-existing memory networks. Further, SWR-related reactivations have been associated with widespread downregulation of hippocampal synapses [112], suggesting a means by which hippocampal representations could be transformed to prune less relevant information while also strengthening specific connections to aid the later reinstatement of relevant information.

Alt-text: Box 3

Thus, we are confronted with two distinct dopamine systems, and we propose that each system is optimized for the promotion of different memory consolidation processes. Although activation of hippocampal TH^+^ axons coming from either VTA or LC enhances the persistence of memory, LC-TH^+^ neuron activation also enhanced the persistence of other, unrelated memories encoded close in time [28] by globally strengthening the synaptic modifications within the hippocampus [52]. By contrast, VTA-TH^+^ neuron activation enhances hippocampal reactivation [27], a process associated with promoting selective reorganisation of memory traces via systems-level mechanisms (Box 3). Such processes are thought to strengthen connections that directly connect elements of the memory trace encoded in the neocortex and thus reduce its hippocampal dependency 57, 60. It should be noted, however, that the effects of dopamine released from hippocampal LC-TH^+^ axons, in the awake state, on subsequent sleep/rest-associated hippocampal reactivation have not yet been established.

## Different Fates of Novelty-Associated Memories

**Figure 2:**
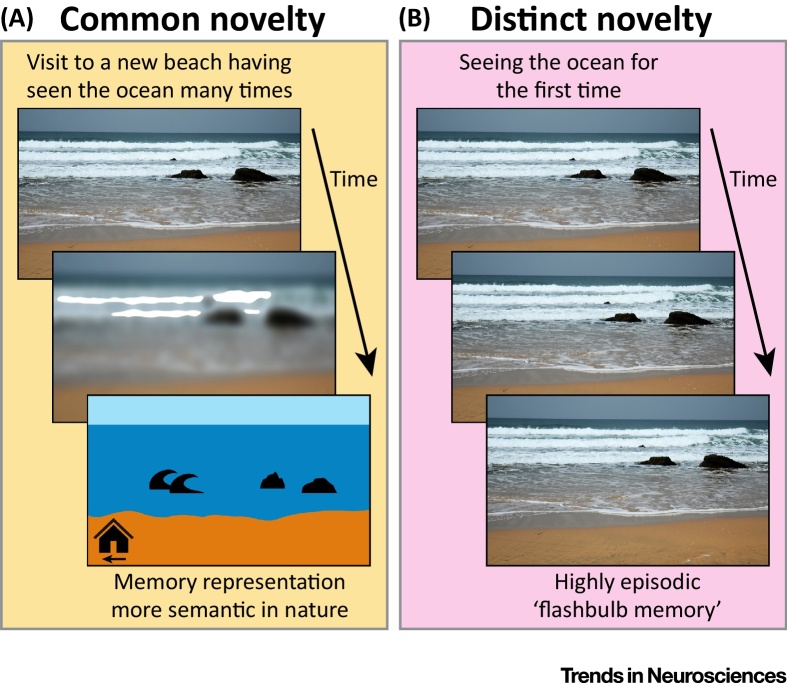
Common and Distinct Novel Experiences May Result in Differences in Memory Fate. (A) A first time visit to a new beach after having seen the ocean many times (common novelty) results in systems memory consolidation and incorporating the memory into pre-existing, neocortical networks (i.e., semantic knowledge). This semantic representation could reflect different aspects of the beach such as the general layout, the quality of the waves, or the way to a nearby bar. (B) By contrast, another person who has never seen the ocean before may retain the memory of a similar experience (e.g., visiting that same beach on the same day) differently. On seeing the vast expanse of the ocean and experiencing the crashing of the waves for the very first time (distinct novelty), they may experience a sense of amazement such that the detailed, hippocampal-dependent episodic memory trace is better retained for a longer time, through stronger initial memory consolidation.

Novelty induces dopamine release in the hippocampus 61, 62 and dopamine-releasing TH^+^ neurons in both VTA and LC increase their activity during exposure to a novel stimulus 27, 28, 45, 63. Moreover, novel experiences promote hippocampal plasticity 23, 42, hippocampal reactivation 27, 64, 65, and memory persistence 10, 12, 28, 50, 66, 67, 68. However, novelty can come in different flavours and can be viewed as a spectrum. On one end of the spectrum are novel experiences that share common aspects with past experiences, such as visiting a new beach after having been to many other beaches before (Figure 2A). We refer to this type of novelty as ‘common novelty’. Common novelty is a novel experience that is similar and relevant to the previous experiences and thus can possibly be memorised by updating the memories already stored in the neocortex. By contrast, completely new experiences such as seeing the ocean for the first time (Figure 2B), pose unique challenges for the brain’s memory systems. Such novelty is by definition unique, bearing little resemblance to previous experiences. Thus, it is less suitable for incorporation into memory representations already stored in the neocortex and it may even interfere with already stored information 69, 70. We refer to this type of novelty as ‘distinct novelty’.

**Figure 3:**
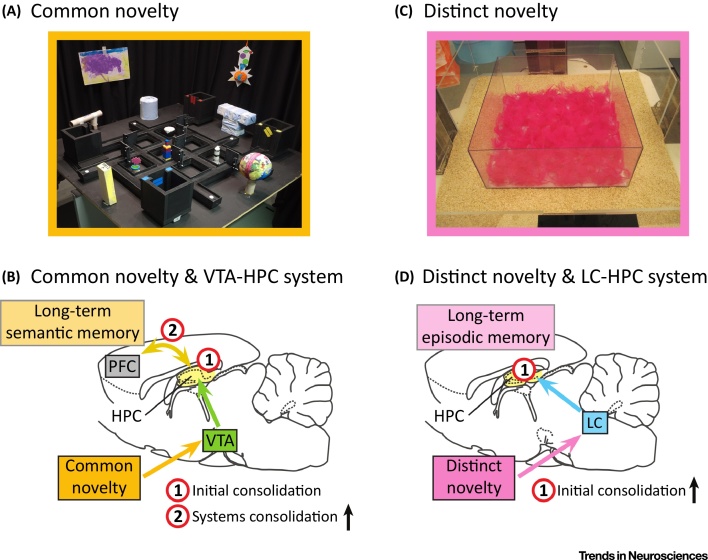
Key Figure: Two Forms of Novelty and Memory Consolidation

A practical example of common novelty in rodent experiments is a new configuration of maze walls, reward location, and orientation of both local and surrounding spatial cues in the crossword maze, for instance (Figure 3A, Key Figure). This represents a highly novel environment that triggers global remapping of the hippocampal cognitive map measured through place cell firing [27]. Still, aspects of the task and the surroundings have been experienced before, thus providing a cognitive substrate for the retention of the new experience. In these experiments, optogenetic activation of hippocampal VTA-TH^+^ axons was sufficient to boost memory retention in a retrieval test held 1 h later. A possible contributing factor to this effect is dopaminergic modulation of synaptic plasticity 23, 71, to trigger initial memory consolidation in the hippocampus. On a network level, optogenetic activation of hippocampal VTA-TH^+^ axons enhanced SWR-associated memory reactivation in the hippocampus in a dopamine-dependent manner [27]. Thus, activation of the hippocampal VTA-TH^+^ axons during encoding resulted in enhanced memory persistence accompanied by enhanced SWR-associated reactivation in the intervening period.

SWR-associated memory reactivation is thought to mediate brain-wide systems memory consolidation 53, 57, 58 and is particularly important for retention of neuronal assembly patterns formed gradually throughout a novel experience [55]. We propose that the VTA-hippocampus system is particularly suited to promote systems memory consolidation while also providing sufficient dopamine to allow initial memory consolidation (Figure 3B). Dopamine release in the prefrontal cortex has been shown to increase hippocampal-prefrontal cortex coherence and subsequent reactivations during sleep, and such extra-hippocampal effects could thus contribute additionally to dopamine’s effect on systems memory consolidation [72]. Activation of the VTA-hippocampus system by common novelty enables the elements of these novel episodes to be incorporated into neocortical semantic knowledge structures based on past experience (i.e., ‘schemas’) 73, 74, and those memories should become less vividly episodic in quality (Figure 2). Moreover, in contrast to the more prominent ‘grace period’ associated with dopamine released by the LC-hippocampal system (which produces a more widespread memory enhancement), this common novelty-associated memory boost involving VTA is more selective to the novel experiences themselves. Data in support of this include an experiment where rats were allowed to explore previously unavailable arms of a radial arm maze (representing new experiences in a known context) [64]. Enhanced reactivation of the novel arm representations was not accompanied by enhanced reactivation of representations associated with familiar arms visited around the same time. Similarly, in crossword maze experiments, enhancement in hippocampal reactivation was limited to spatial maps formed at the time of optogenetic activation of hippocampal VTA-TH^+^ axons and did not enhance reactivation of the maps present in the hippocampal network prior to the optogenetic manipulation [27].

Distinct novelty can come in different forms, such as novel floor substrates as well as objects and spatial context in rodent experiments (Figure 3C), the main commonality being that distinct novelty cannot be related to the animal’s past experiences 28, 75. When animals are given a unique one-of-a-kind experience, this distinct novelty activates the newly-characterised dopaminergic LC-hippocampus system 28, 34. Dopamine coming from hippocampal LC-TH^+^ afferents creates a ‘grace period’ of enhanced initial memory consolidation for events that happened shortly after, as well as before, the event characterised by distinct novelty, supplying the rich contextual details that characterise long-lasting hippocampal-dependent memories 28, 34, 75. Interestingly, a recent study reported that such distinct novelty downregulates the expression of immediate early genes in the prefrontal cortex after learning [75], which suggests that such novel experiences interfere with subsequent systems consolidation processes. Furthermore, distinct novelty causes the memory to be retained for longer in its detailed, hippocampal form and its consolidation is sleep- and thus perhaps hippocampal reactivation-independent [75].

We therefore postulate that the LC-hippocampus system acts to preserve the vivid quality of episodic-like memories by strengthening the hippocampal memory traces through dopamine release and upregulation of initial memory consolidation (Figure 3D). As opposed to becoming more semantic in nature over time, memories consolidated this way retain their rich cooccurring contextual details which were enhanced within the temporal ‘grace period’ and remain anchored to the hippocampus as vivid, flashbulb-like representations 49, 50. Preservation of memories characterised by such distinct novelty in an exceptionally rich, episodic-like form is beneficial due to the inability to assess which aspects of such a salient experience will prove to be the most important [76]. Obviously, the transition between the common and distinct novelty categories is unlikely to be abrupt, but rather gradual, with various novel experiences activating both systems to different degrees depending on the features of the experience.

## Concluding Remarks and Future Perspectives

To summarize, this opinion article proposes that memory of events accompanied by novelty can be selectively retained through either of two distinct dopaminergic mechanisms, depending on the nature of the novel experience itself. In our view, ‘common novelty’ leads to selective enhancement of memory retention via activation of the VTA-hippocampus system, and to initial memory consolidation without a grace period, followed by systems memory consolidation between the hippocampus and neocortex. By contrast, we hypothesise that ‘distinct novelty’ leads to the enhancement and preservation of detailed hippocampus-dependent memory representations in a broader temporal window via greater activation of the LC-hippocampus system. This activation enhances initial memory consolidation in the hippocampus [28], and possibly suppresses systems memory consolidation between the hippocampus and the neocortex 75, 77. Furthermore, hippocampal LC-TH^+^ axons may be better suited for releasing dopamine in response to emotive, infrequently occurring events. The synergistic role of simultaneous noradrenaline and dopamine corelease in promoting plasticity and/or arousal may also be of importance [78]. Put differently, we suggest that the VTA-hippocampus system upregulates memory retention via systems consolidation, ultimately leading to greater memory generalisation (neocortex-dependent long-term semantic memory), whereas the LC-hippocampus system enhances retention in a fashion that serves to more fully preserve the contextual content of the memory (hippocampus-dependent long-term episodic memory). A note should be added in relation to the often discussed roles of VTA and LC in reward signalling and arousal/attention, respectively 3, 79. We would like to emphasise that our postulated roles of these brain regions in memory are separate from and complementary to their abovementioned functions.

Although the involvement of both hippocampal dopaminergic systems in mnemonic processing is well-established, dissecting the qualitative differences between VTA- and LC-mediated memory consolidation requires more systematic testing on both physiological and behavioural levels. Furthermore, while we have discussed the role of VTA and LC activity during actual experiences, their roles in subsequent reactivation events during sleep/rest may also be important and require better understanding. It has been shown that VTA neurons coordinate with hippocampal SWR-associated reactivation during quiet wakefulness but only in some cases during non-REM sleep 80, 81. However, dopamine D_1_/D_5_ receptor activation *in vitro* promotes SWR occurrence [82], and selective closed loop medial forebrain bundle activation (including projections from the VTA) triggered by the activity of a place cell during sleep can create a memory for that location [83]. Additionally, and in line with our hypothesis, SWR-triggered electrical LC activation *in vivo* prevents further SWR occurrence [84], suggesting differential roles for VTA- and LC-hippocampus systems in memory consolidation even during off-line states. For possible differential consolidation mechanisms of these two types of novelty during sleep see [77].

Importantly, since activation of either of the dopaminergic streams during experience leads to a boost in memory retention, future studies contrasting their effects on mnemonic processing should look beyond simple behavioural readout and focus instead on the quality of boosted memory; physiological hallmarks of intra-hippocampal reactivation and hippocampal-neocortical dialogue; and molecular markers of hippocampal and neocortical plasticity 75, 85, 86 (see Outstanding Questions). The recent discoveries regarding the dual nature of dopaminergic modulation of memory consolidation, as discussed in this opinion article, will hopefully inspire future comparative studies that will directly assess the qualitative differences between memory traces consolidated via these two distinct dopaminergic systems.

Outstanding Questions Brain systems that detect either common novelty or distinct novelty: which brain circuits provide information about the different types of novelty to the VTA and LC?

Subcellular localization of dopamine receptors in the hippocampus: what is the subcellular localization pattern of the different dopamine receptor types, particularly D_1_ and D_5_ receptors, in hippocampal principal neurons? Similarly, what is the localization pattern in the various types of interneurons? What are the functional implications of these distinctions?

Innervation pattern of LC-TH^+^ and VTA-TH^+^ axons in the hippocampus and neocortex: what are the specific connectivity patterns of LC-TH^+^ and VTA-TH^+^ axons to principal neurons and various types of interneurons? How do they vary across different species?

Dopamine release in the hippocampus from the two dopamine systems: what are the qualitative and quantitative differences in hippocampal dopamine release from LC-TH^+^ and VTA-TH^+^ axons? What are the specific LC firing patterns that lead to dopamine release from hippocampal LC terminals in natural conditions?

Synergistic role between dopamine and noradrenaline: what are the synergistic interactions between dopamine and noradrenaline released from LC-TH^+^ axons in the hippocampus that may enhance persistence of memory?

LC-hippocampal system and hippocampal reactivation: does dopamine released from hippocampal LC-TH^+^ axons during wake affect subsequent sleep/rest-associated hippocampal reactivation?

VTA-hippocampal and LC-hippocampal systems-mediated memory consolidation: are there qualitative differences between memories consolidated via the VTA and LC dopaminergic systems?

## Glossary

**Dopamine D_1_/D_5_ receptors**: D_1_-like family of receptors, coupled to the G protein G_Sα_ that subsequently activates *de novo* protein synthesis. These receptors are known to gate hippocampal plasticity and memory. In the dorsal hippocampus of mice, the dopamine D_1_ receptor is expressed in granule cells in the dentate gyrus, as well as a subset of inhibitory interneurons in the hilus and CA1/CA3 subregions. Dopamine D_5_ receptor is expressed in pyramidal neurons in CA1 and CA3 and granule cells in the dentate gyrus.
**Flashbulb memory**: a psychological phenomenon whereby unexpected, novel, and often emotionally salient events are remembered for a long time and with unusually high vividness that includes many seemingly unrelated details.
**Hippocampal reactivation**: off-line (i.e., occurring after an actual experience) reoccurrence of network activity patterns in the hippocampus reflecting the cell firing relationships which occurred during the actual experience. Place cell replay after a spatial experience is a well-known example of this phenomenon: as a rodent transverses different place fields during exploration, hippocampal place cells fire in sequences representing current trajectories, and these sequences are then replayed off-line during subsequent sleep/rest episodes. Hippocampal reactivation is thought to play a role in memory consolidation and is prevalent during hippocampal sharp wave-ripple oscillations in non-rapid eye movement (non-REM) sleep.
**Locus coeruleus (LC)**: small nucleus in the brainstem and the main source of noradrenaline in the central nervous system. Noradrenaline released from LC promotes overall arousal and induces attention shifts, but at least some LC neurons are also capable of coreleasing noradrenaline’s precursor dopamine.
**Memory consolidation**: general term for a range of cellular- and systems-level processes that collectively strengthen or modify encoded memories.
**Sharp wave-ripples (SWRs)**: 100–150 Hz oscillation in the hippocampus (sharp wave in the stratum radiatum, ripple in the pyramidal layer) occurring during awake behaviour and especially during rest and sleep.
**Tyrosine hydroxylase (TH)**: an enzyme involved in synthesis of catecholamines (dopamine and noradrenaline). In this opinion article, we use the term TH-expressing (TH^+^) neurons to refer to catecholamine-producing neurons in the ventral tegmental area (VTA) and locus coeruleus (LC).
**Ventral tegmental area (VTA)**: midbrain nucleus that is the main origin of dopaminergic fibres innervating the brain’s higher cognitive centres, including the ventral striatum, neocortex, and parts of the limbic system. Dopaminergic neurons in VTA are part of the brain’s reward circuitry, but some are also activated in response to salient stimuli with no immediate reward value.
